# Cortical alterations associated with executive function deficits in youth with a congenital heart defect

**DOI:** 10.1162/imag_a_00371

**Published:** 2024-11-18

**Authors:** Fatme Abboud, Kaitlyn Easson, Melanie Ehrler, Justine Ziolkowski, Charles V. Rohlicek, Bea Latal, Christine Saint-Martin, Guillaume Gilbert, Matthias Greutmann, Gabriel A. Devenyi, Ruth O’Gorman Tuura, M. Mallar Chakravarty, Marie Brossard-Racine

**Affiliations:** Advances in Brain & Child Development Research Laboratory, Centre for Outcomes Research and Evaluation, Research Institute of the McGill University Health Centre, Montreal, QC, Canada; Child Development Center, University Children’s Hospital Zurich, Zurich, Switzerland; Children’s Research Center, University Children’s Hospital Zurich, Zurich, Switzerland; Computational Brain Anatomy Laboratory, Douglas Mental Health University Institute, Montreal, QC, Canada; Cerebral Imaging Centre, Douglas Mental Health University Institute, Verdun, QC, Canada; Department of Pediatrics, Division of Cardiology, McGill University Health Centre, Montreal, QC, Canada; Department of Medical Imaging, Division of Pediatric Radiology, McGill University Health Centre, Montreal, QC, Canada; MR Clinical Science, Philips Healthcare, Mississauga, ON, Canada; Department of Cardiology, University Heart Center, University Hospital Zurich, University of Zurich, Zurich, Switzerland; Department of Psychiatry, McGill University, Montreal, QC, Canada; Center for MR Research, University Children’s Hospital Zurich, Zurich, Switzerland; Department of Biological and Biomedical Engineering, McGill University, Montreal, QC, Canada; School of Physical & Occupational Therapy, McGill University, Montreal, QC, Canada; Department of Pediatrics, Division of Neonatology, Montreal Children’s Hospital, Montreal, QC, Canada

**Keywords:** congenital heart defect, executive functioning, cortical morphometry, non-negative matrix factorization, partial least squares

## Abstract

Adolescents and young adults born with a complex congenital heart defect (CHD) are at risk for executive function (ExF) impairments, which contribute to the psychological and everyday burden of CHD. Cortical dysmaturation has been well described in fetuses and neonates with CHD and early evidence suggests that cortical alterations in thickness, surface area, and gyrification index are non-transient and can be observed in adolescents with CHD. However, cortical alterations have yet to be correlated with ExF deficits in youth with CHD. This study aims to use a data-driven approach to identify the most important cortical features associated with ExF deficits in adolescents and young adults with CHD. To do so, we combined two comparable datasets acquired at the Research Institute of the McGill University Health Centre and the University Children’s Hospital Zurich, each including both youth with CHD and healthy controls. For each participant, a high-resolution T1-weighted magnetic resonance image, a self-reported ExF assessment (the Behaviour Rating Inventory of Executive Function – Adult Scale), and their clinical and demographic characteristics were available. Corticometric Iterative Vertex-Based Estimation of Thickness (CIVET) was used to extract cortical thickness, cortical surface area, and local gyrification index measures. Using orthogonal projective non-negative matrix factorization (OPNMF), we identified non-overlapping spatial components that integrate cortical thickness, cortical surface area, and local gyrification index and capture structural covariance across these features. Behavioral partial least squares correlation (bPLS) analysis was then used to compute correlations between the individual variability in the OPNMF covariance patterns and ExF outcomes for each subject. A total of 56 youth with CHD who underwent cardiopulmonary bypass surgery before 3 years of age and 56 age- and sex-matched healthy controls were included in our analyses. Cortical grey matter volume, cortical thickness, and cortical surface area were found to be significantly reduced in CHD patients compared to controls. OPNMF identified 12 stable cortex-wide components summarizing the inter-subject variability in cortical thickness, cortical surface area, and local gyrification index. bPLS revealed two significant latent variables (LV) accounting for a total of 82.8% of the variance in the sample, each describing distinct patterns between the brain and cognitive data. LV1 summarized a pattern of belonging to the CHD group, worse scores on most Behaviour Rating Inventory of Executive Function – Adult Scale (BRIEF-A) scales, younger age at MRI, and female sex. This pattern was associated with increased cortical thickness, local gyrification index, and decreased cortical surface area in several OPNMF components. Finally, we identified a positive relationship between the LV1 brain-behavior pattern and total aortic cross-clamp time in the CHD group, indicating that longer aortic cross-clamp time was associated with worse neuropsychological outcomes. In this study, we uncover novel multivariate relationships between ExF and alterations in cortical thickness, surface area, and local gyrification index in adolescents and young adults with CHD using a data-driven approach. Although our findings highlight the important role played by the cortex in higher-order cognitive processes, future studies are needed to elucidate the individual contribution of individual and clinical attributes into the deficits observed in this population.

## 1 Introduction

Congenital heart defects (CHD) are the most common neonatal birth defects, affecting approximately 0.8% of live births, and are one of the leading causes of infant mortality (Bernier et al., 2010;Zimmerman et al., 2020). While advancements in healthcare have substantially increased survival age to throughout adulthood, it is now well recognized that many adolescents and adults with complex CHD will experience psychosocial and higher-order cognitive difficulties (Kovacs et al., 2022;Perrotta et al., 2020). Executive function (ExF) deficits are hallmarks of the cognitive challenges experienced by adolescents and adults with CHD (Calderon & Bellinger, 2015;Klouda et al., 2017;Marelli et al., 2016;Perrotta et al., 2020). ExF refers to a group of higher-order cognitive processes that are essential for self-regulation and behavioral control. ExF deficits may also negatively affect academic achievement, employability, and psychological well-being (Calderon & Bellinger, 2015). In survivors of CHD, worse ExF has been found to be associated with male sex, increased CHD severity, and poorer socioeconomic status (Cassidy et al., 2015;Klouda et al., 2017;Majeed et al., 2022;Sanz et al., 2017;Schlosser et al., 2022;Tyagi et al., 2017). While ExF deficits have been well characterized in the CHD population, the neural correlates of these deficits remain to be comprehensively assessed.

Several structure-function studies have found total and regional brain volume, sulcal pattern similarity, and white matter microstructural integrity to be associated with better ExF outcomes in adolescents and adults with CHD (Brossard-Racine & Panigrahy, 2023). Cortical dysmaturation, including reduction in cortical grey matter volume, surface area, and altered cortical folding, has been well documented in fetuses and infants with CHD (Bonthrone et al., 2021;Clouchoux et al., 2013;Kelly et al., 2017;C. Ortinau et al., 2013;C. M. Ortinau et al., 2019;Schellen et al., 2015;Skotting et al., 2021). However, they have not been extensively studied in older individuals with CHD. In adolescents with CHD, cortical grey matter volume was found to be significantly reduced when compared to healthy peers (von Rhein et al., 2014;Watson et al., 2016). Other studies assessing the more subtle features of the cerebral cortex in adolescents and adults with CHD, such as cortical thickness, cortical surface area, and gyrification, are even more scarce reporting sometimes divergent findings with little anatomical specificity or overlap (Cordina et al., 2014;von Rhein et al., 2014;Watson et al., 2016).

Recent studies have identified that sulcal pattern similarity in the left temporal and frontal lobes as well as the right and left hemispheres is associated with better ExF in adolescents and adults with CHD (Asschenfeldt et al., 2020;Morton et al., 2020,^2021^). Nevertheless, many other cortical features, such as cortical thickness, cortical surface area, and gyrification, that have been associated with better ExF in other healthy and clinical populations, remain to be evaluated as possible correlates of ExF in individuals with CHD (Østgård et al., 2016;Sasabayashi et al., 2017;Tamnes et al., 2010).

Prior research efforts have generally overlooked the complex, inter-dependent relationship between cortical features and the interrelatedness of cortical regions when evaluating their association with ExF outcomes. Multivariate analytical frameworks that integrate multiple cortical features, including cortical thickness, cortical surface area, and local gyrification index, have recently been used to examine cortical development in healthy individuals and have highlighted associations between structural covariance patterns and demographics and cognitive ability (Kalantar-Hormozi et al., 2023). Structural covariance in the human cortex has been shown to give rise to higher-order cognitive functions such as ExF in previous studies (Mechelli et al., 2005). Moreover, the analytical framework proposed byKalantar-Hormozi et al. (2023)has the additional benefit of preserving individual variability, which is critical when working with a heterogenous population such as CHD, where both cognitive outcomes and brain alterations exist on a spectrum. Therefore, the main objective of this study is to identify the cortical correlates of ExF deficits in adolescents and young adults with CHD. To do so, we choose to follow methodology similar to that proposed byKalantar-Hormozi et al. (2023)to integrate MRI-derived measures of cortical thickness, cortical surface area, and local gyrification index and identify patterns of structural covariance using orthogonal projective non-negative matrix factorization (OPNMF). This allows us to account for the interdependent relationship between cortical features. Furthermore, we utilize behavioral partial least squares correlation (bPLS) to identify associations between structural covariance patterns and ExF.

## 2 Methods

### 2.1 Participants

A total of 128 participants with CHD (49.2% female) aged 16 to 32 years and 112 healthy controls (54.4% female) enrolled as part of two parallel case-control studies, one at the Research Institute of the McGill University Health Centre (MUHC) and the second at University Children’s Hospital Zurich (UCHZ), were considered for this study. The recruitment and enrollment procedures have been previously described (Ehrler et al., 2020;Fontes et al., 2019). Participants from these two cohorts were included in the current study if they had completed a high-resolution T1-weighted structural MRI and had available data on self-reported ExF. Only term-born participants (born >36 weeks of gestation) who underwent open-heart surgery using cardiopulmonary bypass before 3.0 years of age and who did not present with genetic abnormalities or intellectual disability (IQ < 70) were included in the CHD group in the current study. Healthy controls were excluded if they presented with any brain malformation, neurologic or neurodevelopmental conditions. From the original available datasets, a total of 75 participants (MUHC:15; UCHZ:60) had to be excluded as they did not meet the above criteria. Therefore, the sample proceeding to further analyses consisted of 62 adolescents and young adults with CHD and 102 healthy controls. Considering that the combined dataset consisted of a disproportionate number of controls to CHD participants, we implemented a matching method following exclusions due to quality control or processing failures to ensure that our analyses were not driven by the larger control group. Using the R (v4.0.3) (R Core Team, 2020) package MatchIt v4.5.0 (Ho et al., 2011), we matched healthy controls to CHD participants based on age and sex using Euclidean distance to compute the distance metrics and nearest neighbor method to identify matches.

This study was approved by the Pediatric Research Ethics Board of the MUHC and the ethical committee of the canton of Zurich in Switzerland. Written informed consent was collected from participants or legal guardians if they were younger than 18 years of age.

### 2.2 Executive function

To assess ExF, the Behaviour Rating Inventory of Executive Function— Adult Scale (BRIEF-A) was completed by all participants at both centers (Rouel et al., 2016). Participants from the UCHZ completed the German version of the BRIEF-A (Roth & Gioia, 2005). The BRIEF-A is a norm-referenced questionnaire that assesses ExF in everyday life and includes nine subscales: inhibit, shift, emotional control, self-monitor, initiate, working memory, plan and organize, task monitor, and organization of materials. These subscales can be combined to provide two summary scales: the behavior regulation index and metacognition index summary scores. The sum of all raw subscales provides the global composite score. Higher T-scores on the BRIEF-A indicate worse functioning and T-scores greater than or equal to 65 on any scales or indices are considered clinically abnormal (Roth & Gioia, 2005).

### 2.3 Individual and clinical variables

Clinical information that pertains to the ante, intra, and postsurgical period was collected from medical records for the CHD participants. The relevant clinical variables examined in the current study include CHD physiology (i.e., single or two-ventricle), the number of open-heart surgeries, age at first open-heart surgery, and total aortic cross-clamp time. We collected maternal education as a four-level ordinal categorical variable, with 1 being the lowest possible education level (elementary school or less) and 4 being the highest possible education level (standard College of General and Professional Teaching (CEGEP), college, or university education) as maternal education has been shown to be a reliable proxy of socioeconomic status (Asztalos et al., 2017). This scale was then dichotomized as high versus low, distinguishing maternal education that referred to a completed CEGEP, college, or university degree from maternal education lower than completed CEGEP or college, and was included in further multivariate analyses to facilitate the interpretation of the findings. Participant level of education was collected on a 5-level scale that recorded current or highest completed level of education depending on if participants were students or had graduated at the time of assessment.

### 2.4 MRI acquisition and processing

Participants from the MUHC underwent a brain MRI on a clinical 3.0 Tesla MRI system (Achieva X, Philips Healthcare, Best, The Netherlands). Three-dimensional T1-weighted images were acquired using a magnetization-prepared spoiled gradient-echo sequence (TE = 3.7 ms, TR = 8.1 ms, TI = 1,010 ms, shot interval = 3,000 ms, voxel size = 1.00 × 1.00 × 1.00 mm^3^, FOV = 240 × 240 × 180 mm, flip angle = 8°) using a 32-channel head coil.

Participants from the UCHZ also underwent a brain MRI on a clinical 3.0T MRI system (MR750, GE Healthcare, Waukesha, WI, United States). Three-dimensional T1-weighted images were acquired (TE = 5 ms, TR = 11 ms, TI = 600 ms, voxel size = 1.00 × 1.00 × 1.00 mm^3^, FOV = 256 x 256 x 154 mm^3^, flip angle = 8°) using a three-dimensional spoiled gradient echo (SPGR) pulse sequence. All images were then clinically reviewed for brain abnormalities by an experienced neuroradiologist who was blinded to the participants’ medical history at both centers. We categorized MRI abnormalities of potential clinical significance into two categories: 1) focal and multifocal abnormalities (e.g., gray matter heterotopia, white matter lesion/injury, focal infarction) and 2) global abnormalities (e.g., developmental anomaly, enlarged or asymmetrical ventricles). The range of abnormalities detected is reported inSupplementary Section 1.1. Raw images were visually inspected for any motion artifacts and processing quality prior to any other processing. Images were then preprocessed using the iterativeN3 preprocessing pipeline (https://github.com/CoBrALab/iterativeN3), which standardizes T1-weighted images by performing contrast inhomogeneity correction, intensity normalization, and masking the brain from non-brain tissue (e.g., neck and skull). Output images were then assessed for quality via visual inspection; any outputs that failed were not included in subsequent steps.

To acquire measures of cortical grey matter volume, cortical thickness, and cortical surface area, the corticometric iterative vertex-based estimation of thickness (CIVET; v2.1.1) processing pipeline was used (Ad-Dab’bagh et al., 2006;Lepage et al., 2017;A. P. Zijdenbos et al., 2002). Local gyrification index was estimated using the surface ratio method (Toro et al., 2008) and the CIVET pial surface (For details, seeSupplementary Section 1.2). CIVET provides estimates of the cortical features at a total of 81,924 vertices across both hemispheres. Midline vertices were masked as cortical thickness and cortical surface area estimates in this region are unreliable. Therefore, the final analyses were performed across 77,122 vertices. Cortical feature extraction was performed on the CBRAIN platform (Sherif et al., 2014).

### 2.5 Harmonization

Following cortical feature extraction and participant matching, cortical grey matter volume, cortical thickness, cortical surface area, and local gyrification index were then harmonized to minimize the effects of scanner variability due to the multi-site nature of this study using CovBat (https://github.com/andy1764/CovBat_Harmonization). CovBat is a batch-correction tool that was applied to the cortical features to harmonize their mean, variance, and covariance across the two batches (Chen et al., 2019,^2022^;Fortin et al., 2017,^2018^;Johnson et al., 2007). For each of the four cortical features, a vertex-wise matrix was built where each row contained the cortical measure at each vertex, and each column contained all the cortical vertices of each subject. The four matrices were then concatenated vertically to form a vertex-by-subject matrix, which was then submitted to CovBat for batch correction in order to remove batch effects from the combined covariance of the four cortical features (Ziolkowski, 2022). Variability due to age, sex, and group status was preserved. The output harmonized vertex-by-subject matrix was then split to obtain four matrices containing each CovBat-corrected cortical feature. CovBat correction was performed in Python v3.9.7 (http://www.python.org). (For details on harmonization validation, seeSupplementary Section 1.3.)

### 2.6 Statistical analysis

Descriptive statistics were first used to characterize the sample, and Shapiro–Wilk tests and normal quantile–quantile plots were used to assess the normality of variables. Group comparisons for participant characteristics and cortical features were assessed using analysis of covariance (ANCOVA) for continuous variables and Pearson χ^2^or Fisher exact tests for categorical variables as appropriate. ANCOVA was used to control for potential confounders, including age, sex, and maternal education, when assessing group differences in BRIEF-A scores and cortical features. When the normality assumption was violated, Mann-Whitney U tests or log-transformed variables were used as appropriate. p-values less than 0.05 were considered significant. All descriptive statistics were performed in R v4.0.3. (R Core Team, 2020).

#### 2.6.1 Orthogonal projective non-negative matrix factorization

Our analysis workflow closely followed that ofKalantar-Hormozi et al. (2023). First, to identify brain regions where inter-subject variability may exist in the examined cortical features and capture structural covariance, orthogonal projective non-negative matrix factorization (OPNMF) was employed (Lee & Seung, 1999;Patel et al., 2020,^2022^;Sotiras et al., 2015). OPNMF approximates a decomposition of a given input matrix (V; m x n) into a component matrix (W; m x k) and weight matrix (H; k x n) such that every column of V can be recomputed as a linear combination of the columns in W using the coefficients (weights) supplied by the columns of H. The orthogonality constraint ensures that identified components are minimally overlapping and encourages sparsity in the solution. The result essentially identifies distinct brain regions, and helps with interpretation of the findings (Sotiras et al., 2015). Additionally, the non-negativity constraint of OPNMF ensures the data are represented in an additive and parts-based manner (Lee & Seung, 1999). The publicly available code athttps://github.com/asotiras/brainpartswas used to perform the OPNMF with Octave (https://www.gnu.org/software/octave/doc/v5.2.0/). OPNMF was initialized with a non-negative double singular value decomposition (SVD) with a maximum iteration of 100,000 and tolerance = 0.00001, as described byKalantar-Hormozi et al. (2023).

Prior to submitting the input matrix into OPNMF, vertex-wise CovBat corrected cortical thickness, cortical surface area, and local gyrification index were residualized for CovBat corrected total cortical grey matter volume to ensure that any variability observed in the cortical features is independent of variability in total cortical grey matter volume across individuals. The three matrices of cortical thickness, cortical surface area, and local gyrification index were then concatenated to form the input matrix consisting of rows of vertices and columns of subject-metric combinations. The input matrix was then normalized using a z-scoring method on a per vertex basis as all structural metrics exist in scales of varying magnitude. To ensure non-negativity of the matrix, resulting z-scored metrics were shifted by the minimum z-scored value. The OPNMF input was prepared using the publicly available code athttps://github.com/CoBrALab/cobra-nmf/tree/main/vertex.

To identify the optimal number of components, split-half stability analysis was carried out. To do so, the change in spatial stability and reconstruction accuracy of the decomposition was examined as the number of components increased, as previously described byKalantar-Hormozi et al. (2023),Patel et al. (2020,^2022^), andRobert et al. (2022). OPNMF was applied separately to a random two-group split of the participants, and the output similarity was compared to assess the spatial stability. The gain in accuracy was then estimated as we increased the number of components by observing the change in reconstruction error. A decomposition was considered optimal if it had high spatial stability and low reconstruction error with minimal complexity (i.e., lower number of components).

#### 2.6.2 Behavioral partial least squares correlation analysis

To establish cortical features that covary with measures of ExF and participant characteristics, we implemented a behavioral partial least squares correlation (bPLS) model. bPLS is a multivariate analysis technique that can identify patterns of covariance between two sets of variables by applying SVD (Krishnan et al., 2011;McIntosh & Lobaugh, 2004). The output of bPLS is a set of latent variables (LVs) that describe the patterns of association between the cortical features and participant characteristics which maximally covary. bPLS was performed using the Python package pyls v0.0.1 (https://github.com/rmarkello/pyls).

The input to bPLS was a set of two matrices. The first contained the brain data in the form of the component subject-metric weights obtained during the OPNMF run, while the second, a participant characteristics matrix, contained the following information: disease status (coded as CHD = 1, no CHD = 0), age in years, sex (coded as female = 1, male = 0), the presence of brain lesions (coded as yes =1, no=0), maternal education (coded as high level = 1, low level = 0), and each of the BRIEF-A summary and subscales (continuous scores). Permutation testing, bootstrap resampling, and split-half stability were performed to assess significance and generalizability of results (For details, seeSupplementary Section 1.4).

Although bPLS patterns were derived from analyses integrating both the CHD and the control groups, we were interested in investigating whether variations within the bPLS patterns in the CHD group aligned with clinical variables. As such, we additionally performed exploratory analyses to identify relationships between the participant expression of the brain (x-scores) and behavior (y-scores) patterns captured by the bPLS LVs and clinical and individual variables in the CHD participants. We performed Spearman’s rank-order correlation tests for continuous variables (age at first surgery and total aortic cross-clamp time). Associations with categorical clinical variables (CHD physiology and number of cardiopulmonary bypass surgeries) were not explored due to reduced distribution of the number of participants within the categories. p-values less than 0.05 were considered significant. Exploratory analyses were performed in R v4.0.3 (R Core Team, 2020).

## 3 Results

### 3.1 Participant characteristics

**Fig. 1.**
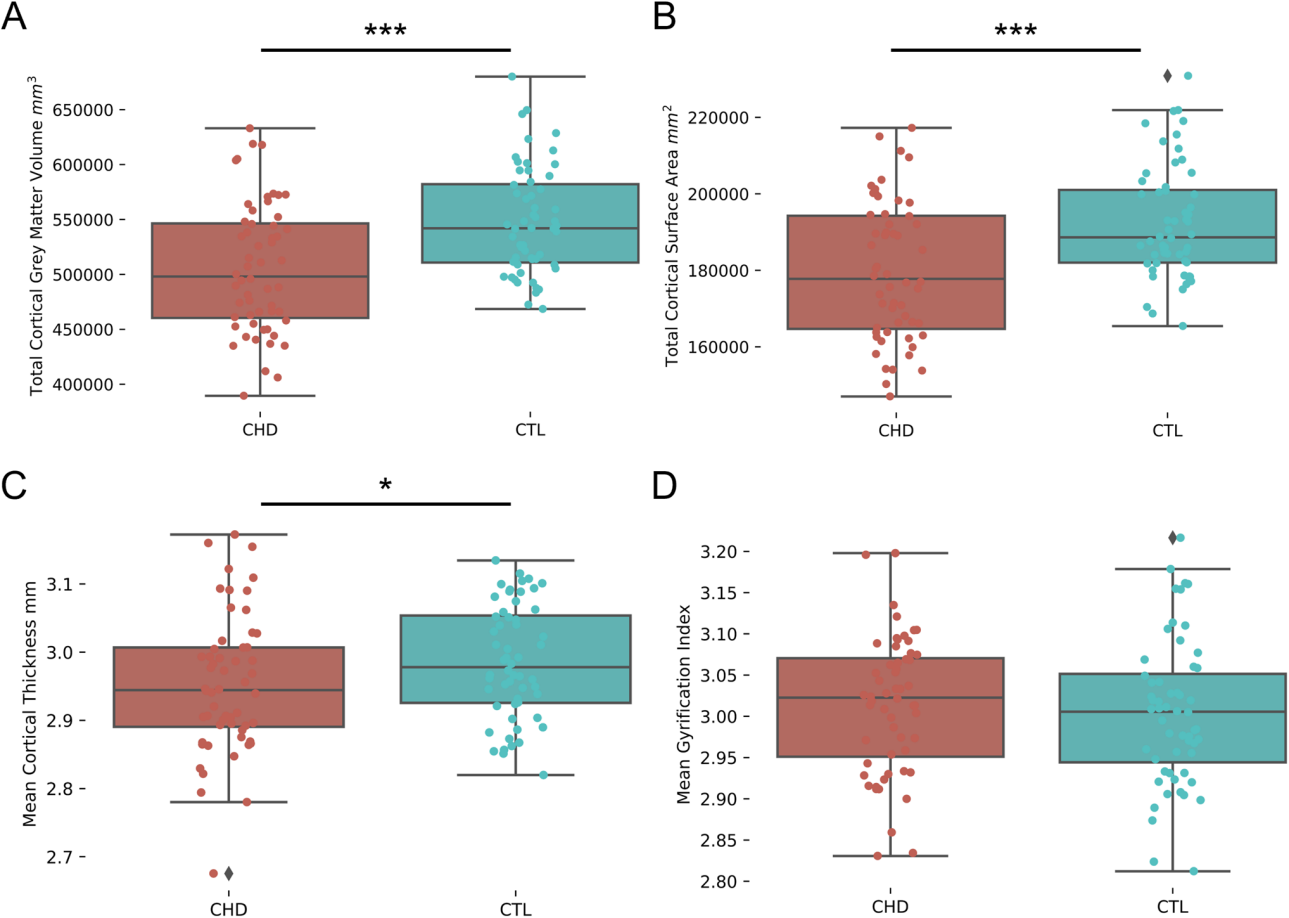
Harmonized cortical features examined in the current study. (A) Total cortical grey matter volume (mm^3^), (B) total cortical surface area (mm^2^), (C) mean cortical thickness (mm), and (D) mean gyrification index. *p < 0.05, ***p < 0.001.

A total of 13 participants were excluded for having not passed raw (n = 1) or post-processing (n = 12) image quality control. Following all exclusions and participant matching, the final dataset consisted of 56 participants with CHD (mean age 21.4 years) and 56 healthy controls (mean age 21.6 years) (For a complete sample breakdown, seeSupplementary Fig. 1). Matched participants did not differ from the unmatched participants with respect to sex, maternal education, or performance on the BRIEF-A. The CHD and control groups did not differ with respect to maternal education; however, the participant level of education was significantly lower in the CHD group when compared to controls, with more healthy controls having completed or being in the process of completing a university degree than participants with CHD. A significantly greater proportion of CHD participants presented with MRI brain abnormalities than controls (p < 0.001;Supplementary Table 1). Participant characteristics are presented inTable 1.

**Table 1.** Summary of participant individual and clinical characteristics.

Mean ± SD, n (%), median [range]	Categories	CHD (n = 56)	Control (n = 56)	p-value
Age at MRI (years)		21.4 ± 3.9	21.6 ± 3.7	0.692
Sex	Female	27 (48.2)	27 (48.2)	1
	Male	29 (51.8)	29 (51.8)	
Maternal highest completed education level ^a^	Elementary school (6 ^th^ grade) or less	1 (1.8)	0 (0)	0.059
	High school (partially completed or graduated)	11 (19.6)	3 (5.4)	
	CEGEP/college partially completed (at least one year) or specialized training (e.g., apprenticeship)	16 (28.6)	17 (30.4)	
	Standard CEGEP, college, or university education	28 (50)	36 (64.3)	
Participant education level ^b^	High school	11 (19.6)	6 (10.7)	**<0.001**
	Professional school or apprenticeship	16 (28.6)	8 (14.3)	
	CEGEP/College	19 (33.9)	12 (21.4)	
	University education/Technical College (undergraduate or graduate)	9 (16.1)	30 (53.6)	
	Other	1 (1.8)	0 (0)	
CHD physiology	Single Ventricle	8 (14.3)		
	Two-Ventricle	48 (85.7)		
Age at first surgery (months; n = 50)		5.8 ± 8.4		
Number of cardiopulmonary bypass surgeries per individual (n = 52)		1 [1–3]		
Total aortic clamp time (n = 46)		72.7 ± 31.4		
MRI brain abnormalities		18 (32.1)	3 (5.4)	**<0.001**

a One participant with CHD had missing maternal education; as such, we used the median maternal education (standard CEGEP, college, or university education) as an estimate of their maternal education.

b Current or past highest completed education level depending on if participants were students or had graduated at the time of assessment.

CEGEP, College of General and Professional Teaching.

Significant p-values are indicated in bold.

Of the participants with CHD, 48 (85.7%) presented with two-ventricle CHD physiology, including 22 (39.3%) with transposition of the great arteries, 13 (23.2%) with tetralogy of Fallot, 7 (12.5%) with ventricular and atrial septal defects, 2 (3.6%) with double outlet right ventricle, 2 (3.6%) with truncus arteriosus type I, 1 (1.8%) with total anomalous pulmonary venous connection, and 1 (1.8%) with coarctation of the aorta. Single-ventricle physiology was present in 8 (14.3%) participants and included 3 (5.4%) with pulmonary atresia, 1 (1.8%) with Ebstein’s pulmonary atresia, 1 (1.8%) with double inlet left ventricle, 1 (1.8%) with hypoplastic left heart syndrome, 1 (1.8%) with tricuspid atresia, and 1 (1.8%) with unspecified single-ventricle physiology.

### 3.2 Executive function

Controlling for age, sex, and maternal education, mean scores were significantly higher on 9 out of 12 BRIEF-A summaries and subscales, indicating worse performance in the CHD group compared to the control group. Moreover, a greater proportion of individuals with CHD (12.5–28.6%) performed below the cut-off for clinically abnormal ExF as compared to controls (0–12.5%). The BRIEF-A results are presented inTable 2.

**Table 2.** Comparison of mean outcome scores on the Behaviour Rating Inventory of Executive Function-Adult scale (BRIEF-A) in adolescents and young adults with CHD and healthy controls.

Outcomes	CHD Mean ± SD	Control Mean ± SD	p-value	CHD Abnormal n (%)	Control Abnormal n (%)	p-value
Behavioral Regulation Index	53.9 ± 11.0	48.4 ± 8.1	**0.001**	9 (16.1)	4 (7.1)	0.237
Inhibit	53.3 ± 11.2	49.9 ± 8.3	**0.049**	10 (17.9)	1 (1.8)	**0.008**
Shift	53.4 ± 12.9	50.7 ± 10.2	0.17	8 (14.9)	5 (8.9)	0.557
Emotional Control	53.6 ± 10.7	48.5 ± 9.7	**0.004**	9 (16.1)	4 (7.1)	0.237
Self-Monitor	52.7 ± 11.8	46.2 ± 8.1	**<0.001**	9 (19.1)	1 (1.8)	**0.016**
Metacognition Index	55.0 ± 10.9	50.0 ± 8.0	**0.007**	11 (19.6)	3 (5.4)	**0.042**
Initiate	53.8 ± 11.7	50.9 ± 8.8	0.152	9 (16.1)	6 (10.7)	0.580
Working Memory	56.2 ± 11.6	51.9 ± 9.4	**0.031**	16 (28.6)	5 (8.9)	**0.014**
Plan and Organize	52.9 ± 9.5	49.4 ± 7.3	**0.035**	7 (12.5)	3 (5.4)	0.321
Task Monitor	55.8 ± 10.9	52.9 ± 10.4	0.050	11 (19.6)	7 (12.5)	0.441
Organization of materials	53.1 ± 12.0	46.2 ± 7.4	**0.001**	12 (21.4)	0 (0)	**< 0.001**
Global Executive Composite	54.9 ± 11.1	49.1 ± 7.5	**0.001**	13 (23.2)	2 (3.6)	**0.004**

Significant p-values are indicated in bold.

### 3.3 Cortical features

Adolescents and young adults with CHD presented with significantly lower total cortical grey matter volume (F(1,107) = 26.78, p < 0.001) and mean cortical thickness (F(1,107) = 5.87, p = 0.017) and cortical surface area (F(1,107) = 23.46, p < 0.001) than healthy controls when controlling for age, sex, and maternal education. Mean local gyrification index did not differ significantly between the two groups (F(1,107) = 0.32, p = 0.57). Boxplots of cortical features can be seen inFigure 1. Univariate analyses between cortical features and ExF can be found inSupplementary Section 1.5.

### 3.4 Orthogonal projective non-negative matrix factorization

The input matrix to OPNMF consisted of the harmonized cortical thickness, cortical surface area, and local gyrification index measurements of all subjects residualized for cortical grey matter volume. The optimal number of components that balanced both stability and reconstruction error was identified to be 12 through stability analysis (Supplementary Fig. 2). The cortical mappings of the spatial components can be seen inFigure 2. The following are descriptions of each identified component:

**Fig. 2.**
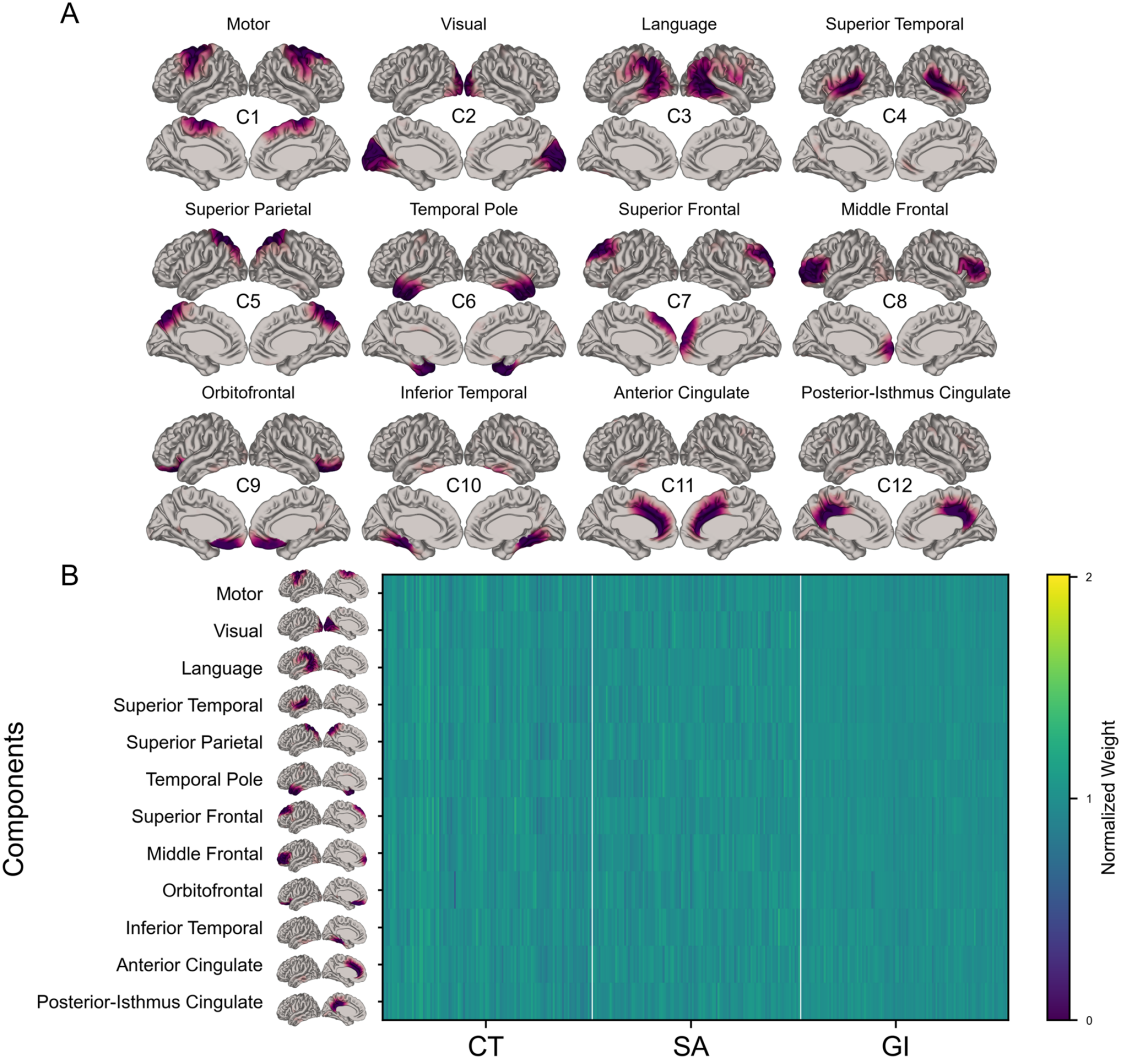
(A) Lateral and medial views of the left and right hemispheres of the 12 components identified through orthogonal projective non-negative matrix factorization (OPNMF). Darker regions on the cortical mappings signify vertices that load more heavily onto the component. (B) OPNMF normalized weight matrix highlighting the subject-specific weightings for each of the 12 components. Matrix was normalized for visualization purposes only. Distributions of un-normalized OPNMF weights for each metric are provided inSupplementary Figure 3. CT, cortical thickness; SA, cortical surface area; GI, local gyrification index.

1) Component 1 (Motor): this component is largely localized to the primary and supplementary motor areas.
2) Component 2 (Visual): strong localization to the visual cortex, encompassing both the primary and secondary visual areas.
3) Component 3 (Language): this component occupies the posterior language area with some extension into the temporal lobe.
4) Component 4 (Superior Temporal): most strongly encompassing the superior temporal gyrus, lateral sulcus, and supramarginal gyrus.
5) Component 5 (Superior Parietal): largely localized in the superior parietal cortex and the more lateral regions of the precuneus.
6) Component 6 (Temporal Pole): the strongest loadings into this component are in the temporal pole with some contributions from the medial temporal areas.
7) Component 7 (Superior Frontal): this component occupies the superior frontal areas bilaterally.
8) Component 8 (Middle Frontal): the middle frontal lobe loads heavily onto this component with some contribution from the left frontal pole only.
9) Component 9 (Orbitofrontal): this component is heavily localized to the lateral and medial orbitofrontal cortex.
10) Component 10 (Inferior Temporal): largely localized in the medial inferior temporal lobe.
11) Component 11 (Anterior Cingulate): this component consists of the anterior cingulate cortex with some extension into superior frontal and paracentral areas.
12) Component 12 (Posterior-Isthmus Cingulate): this component occupies the isthmus cingulate and portions of the posterior cingulate as well as the more medial regions of the precuneus.

### 3.5 Behavioral partial least squares correlation

The weights of the 12 components described above for each participant were subsequently related to participant characteristics and BRIEF-A measures to identify brain-cognition relationships. bPLS analysis revealed two statistically significant LVs each describing a unique relationship between the variables of interest and accounting for a total of 82.8% of the variability in the sample.

**Fig. 3.**
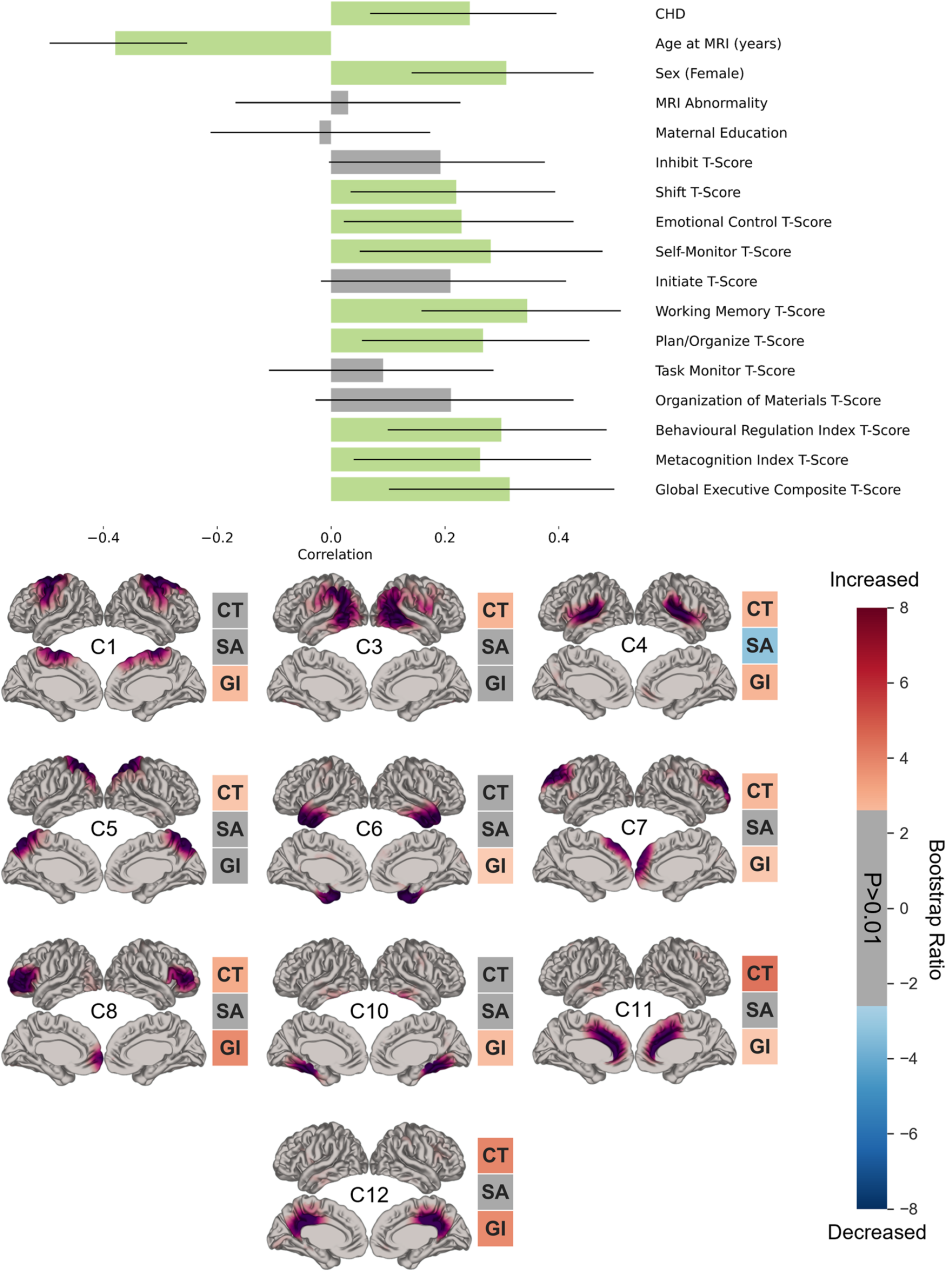
First significant (p < 0.001) latent variable (LV) identified by behavioral partial least squares analysis describing a pattern of belonging to the CHD group and worse performance on 8 scales of the Behaviour Rating Inventory of Executive Function-Adult (BRIEF-A). The bar plot (top) is describing the contribution of participant characteristics and each scale on the BRIEF-A. The x-axis denotes the correlation of each variable in the LV. Error bars are bootstrapped 95% confidence intervals. Green bars indicate variables that are contributing to the LV, and grey bars are variables that do not contribute. Each cortical map (bottom) shows the contribution of each component in the LV. Red indicates an increase of a cortical feature in a given component, and blue indicates a decrease in the cortical feature based on bootstrapping. CT, cortical thickness; SA, cortical surface area; GI, local gyrification index.

The first significant LV (LV1; p = 0.001) directly addressed the primary aim of this study. LV1 accounted for 66.1% of the variance (Fig. 3). The participant characteristics contributing to this LV included: CHD group, younger age at MRI, female sex, and higher scores (indicating worse performance) on 8 out of 12 BRIEF-A scales. These characteristics were associated with increases in cortical thickness in 7 out of 12 components, a decrease in cortical surface area in component 4, and increases in local gyrification index in 8 out of 12 components. The p-values corresponding to the left (brain pattern) and right (behavioral pattern) singular vectors from the split-half analysis are 0.029 and 0.071 respectively.

The second significant LV (LV2; p < 0.001) accounted for 16.7% of the variance. This LV was marked only by younger age at MRI and female sex. These characteristics were associated with increases in cortical thickness across 10 components, decreases in cortical surface area across 3 components, and decreases in local gyrification index across 6 components. The p-values corresponding to the left and right singular vectors are 0.004 and 0.046 respectively. Considering that LV2 did not relate to the primary research question, further analyses were not performed on this LV.

**Fig. 4.**
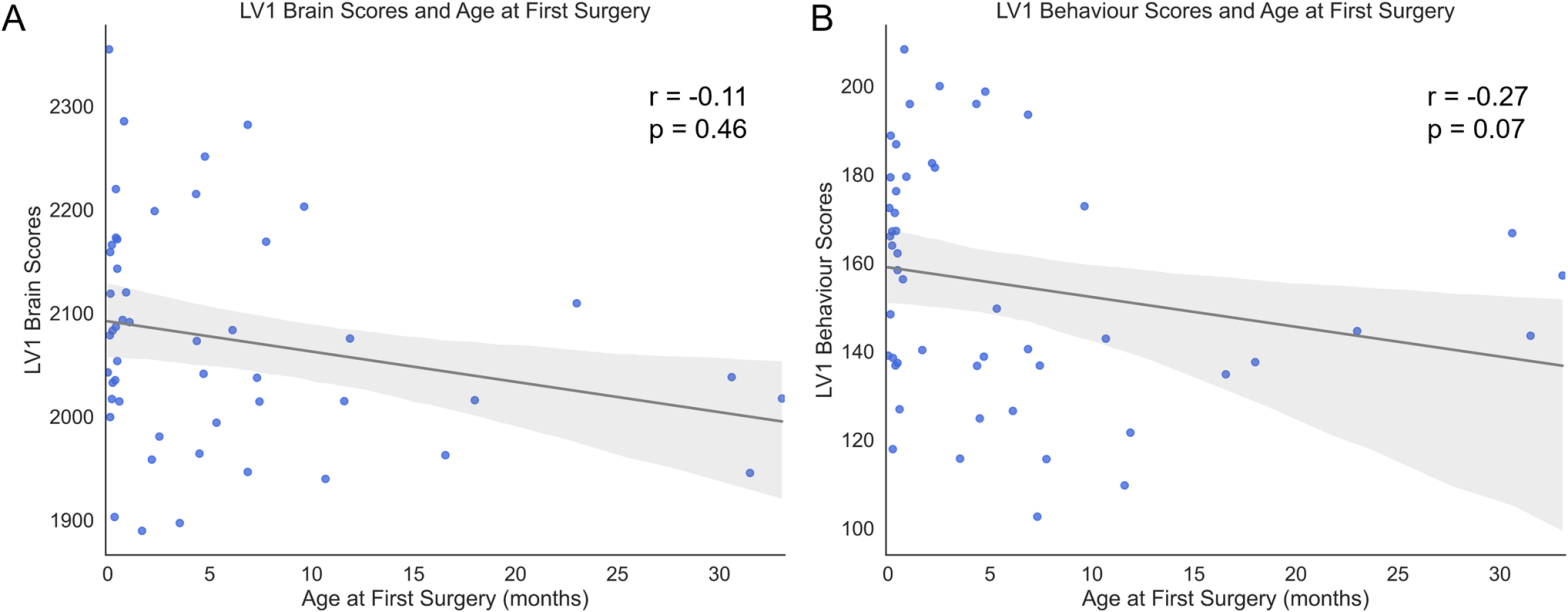
Brain-behavior patterns captured by latent variable 1 (LV1) identified by bPLS and its association with age at first cardiopulmonary bypass surgery. The x-axis denotes the age at first cardiopulmonary bypass surgery in months. The y-axis on (A) denotes the projection of the brain pattern exhibited by LV1 onto the participant data (brain scores). The y-axis on (B) denotes the projection of the behavior pattern exhibited by LV1 onto the participant data (behavior scores).

**Fig. 5.**
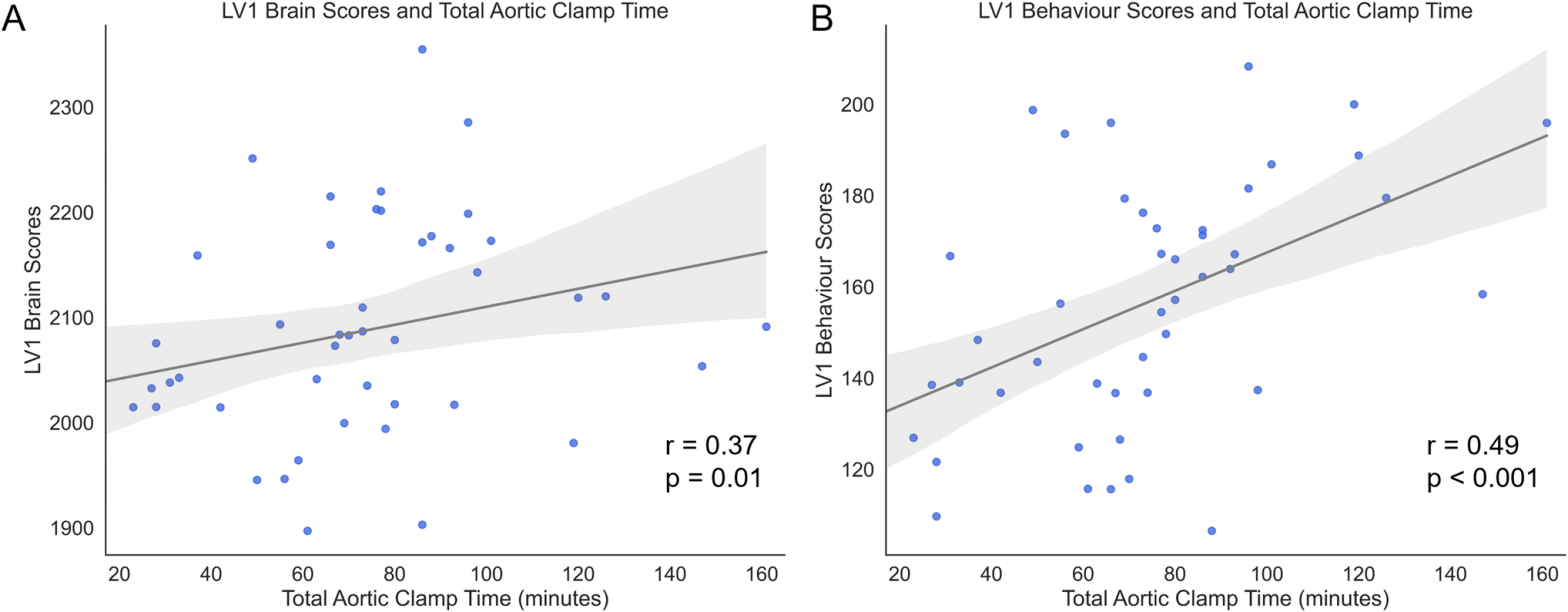
Brain-behavior patterns captured by latent variable 1 (LV1) identified by bPLS and its association with total aortic cross-clamp time. The x-axis denotes the total aortic cross-clamp time in minutes. The y-axis on (A) denotes the projection of the brain pattern exhibited by LV1 onto the participant data (brain scores). The y-axis on (B) denotes the projection of the behavior pattern exhibited by LV1 onto the participant data (behavior scores).

Following the identification of the LVs, we were interested in assessing how clinical variables vary with respect to the brain and behavior patterns described by LV1. LV1 did not capture a consistent relationship between its brain or behavioral scores and age at first surgery (Fig. 4). This was confirmed by the exploratory Spearman’s correlation tests, which showed no significant relationship between the brain or behavioral patterns captured by LV1 and age at first surgery (r = -0.11, p = 0.46; r = -0.27, p = 0.07 respectively). Individuals who spent more time in surgery with a clamped aorta exhibited the brain and behavior pattern captured by LV1 more strongly than those who spent a shorter amount of time with a clamped aorta (r = 0.37, p = 0.01; r = 0.49, p < 0.001 respectively;Fig. 5).

## 4 Discussion

Using a data driven approach, this study identified alterations in cortical thickness, cortical surface area, and local gyrification index on cerebral MRI that are associated with worse ExF in adolescents and young adults with CHD. We found that total cortical grey matter volume, mean cortical thickness, and total cortical surface area were significantly lower in the CHD group compared to the control group when controlling for age, sex, and maternal education. In contrast, mean local gyrification index did not significantly differ between the two groups. Our results converge with the few previous studies that have also observed global reductions in cortical grey matter volume and cortical surface area and no differences in local gyrification index in adolescents and adults with CHD as compared to controls (Morton et al., 2021;von Rhein et al., 2014;Watson et al., 2016). In contrast, previous studies assessing cortical thickness in this population have yielded conflicting results, reporting both widespread cortical thickness reductions in adults with CHD (Cordina et al., 2014) as well as no cortical thickness differences in young adolescents with CHD when compared to healthy controls (von Rhein et al., 2014). The discrepancy in cortical thickness findings may be explained by the differences in participant age and processing techniques between studies.

We discovered two significant LVs using bPLS describing distinct patterns of covariance between the OPNMF weightings of each participant and their characteristics and ExF abilities. The first LV described a pattern of worse performance on 8 out of 12 BRIEF-A subscales, belonging to the CHD group, as well as a substantial contribution of younger age at MRI and female sex. This pattern was associated with marked cortical thickness increases in the precuneus, posterior language, superior temporal, superior parietal, superior frontal, middle frontal, and cingulate regions, as well as reduced cortical surface area in the superior temporal area, and increased local gyrification index in all components except those encompassing the visual, posterior language, superior parietal, and orbitofrontal regions. Several of the identified components contributing to LV1 captured alterations within cortical regions that overlapped with the salience network (SN), default mode network (DMN), and fronto-parietal central executive network (CEN) nodes. This is interesting because EFs are thought to arise through interactions between the SN, DMN, and CEN rather than being mediated by a single cortical network (Menon & D’Esposito, 2022). Moreover, we identified cortical alterations within the lateral sulcus (i.e., component 4), which houses the insular cortex. This region, a key node in the SN, is hypothesized to regulate the activation of the DMN and CEN during stimulus-dependent and independent cognitive tasks and is also known to be structurally connected to nodes within these networks (Molnar-Szakacs & Uddin, 2022;Uddin et al., 2017). LV1 also revealed alterations within the anterior cingulate cortex, an SN node captured by our component 11, which is also thought to control the activity of the CEN, a network involved in goal-directed behavior such as decision-making (Sridharan et al., 2008). Furthermore, alterations were observed in the precuneus, encompassed by both components 5 and 12, which has been identified to be an important structure for cognitive flexibility, monitoring, and task switching (Schroeter et al., 2023;Yeager et al., 2022). Together, these findings suggest that LV1 captured underlying structural networks connecting multiple cortical regions known to be involved in ExF. These findings support the notion that alterations within these interconnected regions in adolescents and young adults with CHD likely drive ExF deficits.

ExF challenges are recurrent issues identified in survivors of CHD, often impacting various facets of the individual’s everyday life. Among these, working memory impairments are the most commonly reported in this population (Asschenfeldt et al., 2020;Badaly et al., 2022;Bellinger et al., 2015;Calderon & Bellinger, 2015;Cassidy et al., 2015;Sanz et al., 2017). When examining the contribution of specific BRIEF-A scores within our model, we found working memory to have the strongest contribution to LV1. Our results are in line with a previous study in typically developing individuals across the lifespan which also observed associations between higher cortical thickness and worse performance on working memory tasks in younger participants (i.e., children, adolescents, and younger adults), and no associations in adults aged 40 years and older (Krogsrud et al., 2021). This is congruent with the pattern observed in LV1 in our sample, which has a significant contribution of younger age at MRI. Although cognitive decline is thought to arise as a consequence of cortical thinning in older adults (Fjell & Walhovd, 2010), the contribution of younger age in the association between higher cortical thickness and ExF deficits may be explained, in part, by the ongoing brain maturation processes still occurring during adolescence and early adulthood. Throughout this period, cortical thinning is thought to reflect ongoing myelination (Natu et al., 2019) and synaptic pruning (Huttenlocher & Dabholkar, 1997). The rate of synaptic pruning is thought to be higher during adolescence in brain regions involved in higher-order cognitive functions, suggesting a critical role of synaptic pruning in the development of ExF (Selemon, 2013). Delayed or reduced synaptic pruning in these regions, as estimated by higher cortical thickness, may be one of the mechanisms that underlie the ExF deficits in CHD. Previous studies in healthy participants have reported associations between higher values of cortical thickness in the temporal and frontal regions, as well as the anterior cingulate cortex, with worse ExF such as cognitive control, working memory, and inhibition in children, adolescents, and young adults which support our hypothesis (Burzynska et al., 2011;Kharitonova et al., 2013;Krogsrud et al., 2021;Squeglia et al., 2013;Tamnes et al., 2010). While the correlation between higher cortical thickness and worse ExF is conflicting with the finding of overall reduced cortical thickness in the CHD group compared to the controls, we believe it is important to note that the bPLS model highlighted increased cortical thickness in specific cortical regions in relation to worse ExF, whereas the group differences do not account for regional variability in cortical features.

While we could not find previous investigations of other cortical features than cortical thickness in relation to ExF in CHD, we could compare our findings to studies in other clinical populations. For instance, in adolescents born preterm, a group that presents with similar patterns of brain and outcome profiles (Easson et al., 2019;Miller & McQuillen, 2007), lower levels of cortical surface area in the temporal areas of the right hemisphere were similarly correlated with worse ExF (Østgård et al., 2016). In young adults with first-episode schizophrenia, higher gyrification index in frontal, parietal, and occipital regions was associated with worse ExF, in line with our findings (Sasabayashi et al., 2017). This overlap across populations suggests a potential common signature of cortical alterations associated with ExF deficits that may not be diagnosis-specific and should be investigated further in future cross-diagnostic studies.

Poorer ExF outcomes in CHD can be driven by several clinical risk factors such as cardiac physiology and CHD severity (Cassidy et al., 2015;Klouda et al., 2017;Sanz et al., 2017;Schlosser et al., 2022;Tyagi et al., 2017). Although we found no relationship between the behavioral pattern of LV1 and age at first surgery, adolescents and young adults with CHD who had longer total aortic cross-clamp time expressed both the brain and behavior patterns captured by LV1 more strongly than those who spent less time on the aortic clamp. Longer cross-clamp time may be indicative of a confounding effect of longer or a greater number of surgical procedures in the presence of more complex cardiac physiology which we were not able to tease out due to the limitations that come with retrospective collection of clinical data.

From a methodological perspective, we want to recognize that although we use the same framework asKalantar-Hormozi et al. (2023), the components identified by OPNMF differed between our two studies. The data-driven nature of OPNMF may give rise to components that are specific to each dataset and depend on a number of technical choices. Our diverging findings withKalantar-Hormozi et al. (2023)may be explained in part in the different normalization methods used between the two studies. Specifically, as our research question sought to address how interindividual differences in cortical covariance patterns impact ExF in CHD, we chose to normalize on a per-vertex basis to ensure that the resulting OPNMF components capture interindividual variability. In comparison,Kalantar-Hormozi et al. (2023)normalized their data across both individuals and vertices which accounts for both inter- and intra-individual variability in the covariance patterns and identifies more metric-specific components. Nevertheless, the components we identified in the current study did overlap qualitatively with components described in another study (Patel et al., 2022) performed in a different sample, suggesting that the components we identified represent nonetheless a stable configuration of the human cortex.

Our findings must be interpreted within the context of the study’s limitations. First, the split-half analysis conducted on LV1 revealed an insignificant p-value (according to a threshold of p < 0.05) for the behavioral pattern of LV1, indicating an unstable LV. As described inSupplementary Section 1.4, the p-value obtained from split-half analysis is calculated by correlating the singular vectors between subsamples of our dataset repeatedly. Considering our total sample of 112 participants (56 with CHD, 56 without), we would expect that analyses performed on half of this sample would not have enough power to capture this pattern. Data-driven approaches benefit from large sample sizes, especially in highly variable populations such as CHD. While the merging of two datasets in this study intended to address this limitation, our rigorous inclusion and exclusion criteria resulted in the exclusion of about half of the individuals from the original datasets. Nonetheless, this study provides compelling evidence of a significant relationship between altered cortical morphometry and ExF deficits. Further validation in larger cohorts may improve the generalizability and robustness of these findings. The clinical and surgical variables included in the current study were extracted from medical charts completed more than 20 years ago; as a result, many participants had missing information. As such, our investigation of the relationships between LV patterns and perioperative variables was only performed on the subsamples with available data for each variable. Finally, due to the low number of participants in some of the categorical clinical variables, we were unable to assess other proxies of severity, such as CHD physiology or the number of cardiopulmonary bypass surgeries. As such, future studies with larger, more diverse participant samples should continue to explore these proxies of severity as potential risk factors for ExF deficits and cortical dysmaturation in youth with CHD.

## 5 Conclusion

This is the first study to use a data-driven approach to determine the contribution of cortical features to ExF variability in adolescents and young adults with CHD. This work reveals a significant relationship between worse ExF and variability in cortical thickness, cortical surface area, and local gyrification index across multiple cortical regions, particularly in the lateral sulcus, the precuneus, and in regions overlapping network nodes known to be involved in ExF. We hope that this study will pave the way for future work investigating the complex roles of the cortex in the functional deficits observed in CHD and emphasize the importance of optimizing care for promoting healthy brain development.

## Supplementary Material

Supplementary Material

## Data Availability

The data was prepared, analyzed, and visualized using publicly available code atgithub.com/CoBrALab/cobra-nmf/tree/main/vertex,github.com/asotiras/brainparts,github.com/alyssadai/nmf_viz. The Pediatric Research Ethics Board of the MUHC and the canton of Zurich in Switzerland prohibit us from making the data publicly available. However, data may be acquired through a formal data-sharing agreement and with appropriate ethics approval.
