# Supplementary material for: Cortical alterations associated with executive function deficits in youth with a congenital heart defect
Source: Imaging Neurosci (Camb). 2024 Nov 18;2:imag-2-00371. doi: 10.1162/imag_a_00371 (PMC12315761; doi:10.1162/imag_a_00371)
Supplement: Supplementary Material [file imag_a_00371-supp.pdf]

Supplementary Information

1.1. MRI brain abnormalities

A significantly greater proportion of CHD participants presented with mild MRI brain abnormalities than controls (p<0.001). Supplementary Table 1 details the potentially clinically significant brain abnormalities observed in this sample.

Supplementary Table 1: Distribution (count) of brain abnormalities detected

	CHD	*Control*
Focal Abnormalities		
Microhemorrhages/Microbleeding	7	-
Focal infarctions or atrophy	3	-
Grey matter heterotopia	3	1
Focal SWI	2	3
Periventricular white matter injury	1	-
Subcortical grey matter lesion	1	-
Global Abnormalities		
Significantly Enlarged Perivascular Spaces	2	-
Cerebellar cortical developmental abnormality[a]	1	-
Chiari 1 malformation	1	-
Total	21	4

[a]Anomaly restricted to cerebellar cortex, a region not included in our analyses

1.2. Extracting Cortical Features

Cortical Grey Matter Volume: CIVET classifies cerebral tissue into white matter, grey matter, and cerebrospinal fluid through a discrete tag point classification method using the INSECT algorithm (Zijdenbos et al., 1998). This is followed by tissue class partial volume evaluation to obtain volume estimations of each tissue type within each voxel (Tohka et al., 2004). Gray matter volume estimates at each vertex were then used for our analyses.

Cortical thickness: cortical thickness was measured as the minimum distance at each vertex between the white and gray matter surfaces (Lerch & Evans, 2005; MacDonald et al., 2000).

A 30mm full-width at half-maximum surface-based diffusion smoothing kernel was used to blur the cortical thickness map (Kim et al., 2005).

Cortical surface area: cortical surface area was calculated using the Voronoi method where the subject area is divided by the model area at each vertex on the surface template between the white and gray matter surfaces on an intermediate tessellated surface mesh (Jubault et al., 2011). A 40mm geodesic surface kernel was used to blur the surface area map (Kim et al., 2005).

Local gyrification index: local gyrification index was calculated using the surface ratio ($SR_x = \frac{surface\ in\ sphere(x,r)}{area\ of\ disc(r)}$) between the pial surface contained within a 20mm sphere around the vertex and the surface area of a circle of the same radius (Toro et al., 2008). The publicly available code at https://github.com/r03ert0/surfaceratio was used to calculate local gyrification index.

1.3. Harmonization validation: methods and results

To ensure that the mean, variance, and covariance of cortical features were indeed harmonized, we conducted a series of validation analyses (Ziolkowski, 2022). We first residualized cortical features before and after CovBat harmonization for age, sex, and disease status to ensure that these variables did not confound the results obtained in the validation process. Validation analyses were conducted using Python v3.9.7.

Mean. Independent two-sample t-tests were conducted to assess differences in mean vertex-wise values of cortical features between batches 1 and 2. Analysis was performed using the SciPy library v1.7.1(Virtanen et al., 2020) for each cortical feature separately at each of the 77122 vertices before and after CovBat correction.

P-values obtained for each of the 77122 vertices were corrected for multiple comparisons using the false discovery rate (FDR) method available in the statsmodels library v0.12.2(Seabold

& Perktold, 2010). The proportion of vertices whose means differed significantly between batches was recorded.

Prior to harmonization, vertex-wise mean measures of cortical thickness, cortical surface area, and local gyrification index differed significantly between batches at 3015-66719 vertices. Following CovBat batch correction, mean values of cortical thickness, cortical surface area, and local gyrification index did not significantly differ between batches at any vertices. Mean cortical grey matter volume did not differ significantly between batches before or after harmonization.

Variance. To validate the CovBat correction of variance, Levene's test function in the SciPy library v1.7.1 was performed on the medians of each cortical feature separately before and after CovBat correction.

Variance was found to be significantly different before harmonization between batches in cortical thickness only ($p < 0.001$). Following harmonization, variance did not differ significantly between sites for cortical thickness ($p = 0.706$). No batch effect was present in cortical grey matter volume, cortical surface area, or local gyrification index variance before or after CovBat harmonization.

Covariance. Principal component analysis (PCA) was performed on the scaled median values of the cortical features using the Scikit-learn library v.3.9.7 (Pedregosa et al., 2011). Independent two-sample t-tests were performed on the principal component (PC) scores to validate the harmonization of covariance across batches.

PCA revealed covariance across cortical features to be significantly different prior to harmonization. T-tests conducted on the PC scores of the four PCs identified by PCA revealed significant batch effects in PC1 ($p<0.01$) and PC2 ($p<0.01$) prior to harmonization but detected no

batch effects in PC3 or PC4. After harmonization, no significant batch effects were identified in any PCs.

1.4. bPLS: assessing significance, contribution, and generalizability

To assess the significance of the resulting LVs obtained from the entire dataset, permutation testing was carried out (Krishnan et al., 2011). To carry out a permutation test, bPLS is applied to a new sample which was obtained by randomly reordering the observations of the matrix containing the brain data while keeping the participant characteristic matrix unchanged. This process was repeated 5000 times to construct a null distribution of singular values against which we can assess the significance of the resulting LVs. We applied a $p < 0.05$ threshold when assessing the significance of the LVs.

To assess the contribution and reliability of each component to each of the resulting LVs, a bootstrap ratio was computed. Using a sampling with replacement method, 5000 random samples were obtained. The bootstrap ratio was then calculated as the ratio between the singular vector weights over the standard error of the bootstrap distribution weight. A BSR threshold of 2.58 was used to assess if brain variables significantly contributed to a given LV, corresponding to a $p < 0.01$ threshold.

Finally, to assess the generalizability of the bPLS algorithm, split-half analysis was implemented. In split-half analysis, the dataset was randomly split into two groups. The original matrices of the left (U) and right (V) singular vectors were projected onto each of the two new samples (to form U1,U2 and V1,V2). The projected matrices were then correlated (i.e., correlate U1 & U2 and V1 & V2) to provide an estimate of the consistency in the discovered LVs. The original observations were permuted, and the process is repeated 200 times to create a null distribution of split-half correlations. A p-value for the left and right singular vectors is calculated

by estimating the probability of surpassing the correlations between singular vectors of the original dataset. Full details on this method can be found at (Kovacevic et al., 2013).

1.5. Univariate correlations between ExF measures and cortical features

For interest, we performed Spearman correlations in R to examine the relationships between our cortical features and ExF measures within each group separately. In the CHD group, we found a positive correlation between mean cortical thickness and Working Memory ($r = 0.34$, $p = 0.0104$), Initiate ($r = 0.29$, $p = 0.031$), and Metacognition Index ($r = 0.28$, $p = 0.036$). We additionally found a negative correlation between mean gyrification index and Initiate ($r = -0.27$, $p = 0.040$) and Emotional Control ($r = -0.27$, $p = 0.046$). However, these correlations do not pass FDR correction. No correlations were identified in the control group.

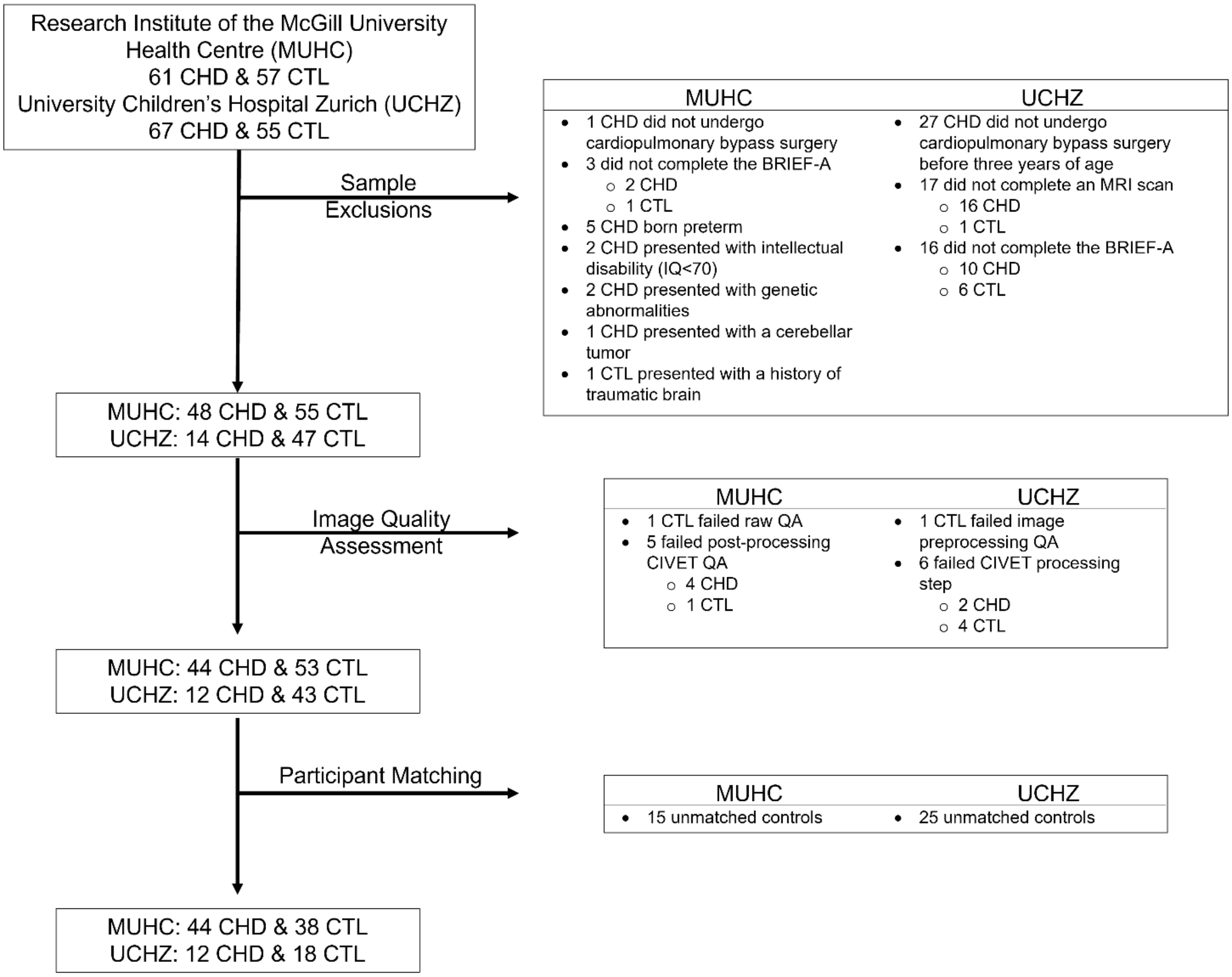

Supplementary Figure 1: Sample breakdown per site. Outline of participant exclusions at each step (CHD: congenital heart defect; CTL; control), including initial exclusions, image quality assessment (QA), and case-control age and sex matching.

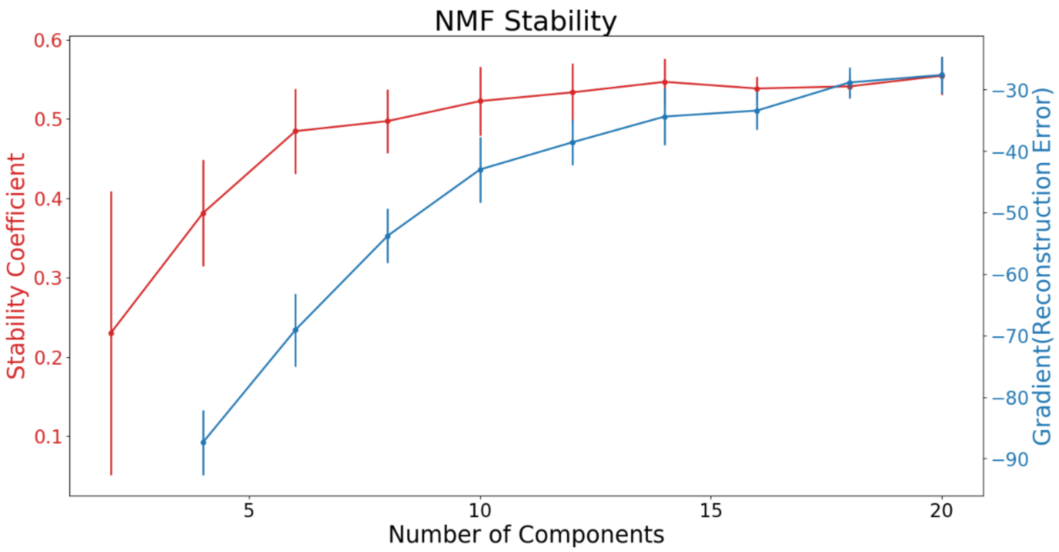

Supplementary Figure 2: Split-half stability analysis results for 2-20 component decompositions. In red, the gain in the stability coefficient as the number of components increases. In blue, the change in the reconstruction error as the number of components increases. It was identified that twelve components provide the optimal balance between stability and accuracy of the decomposition.

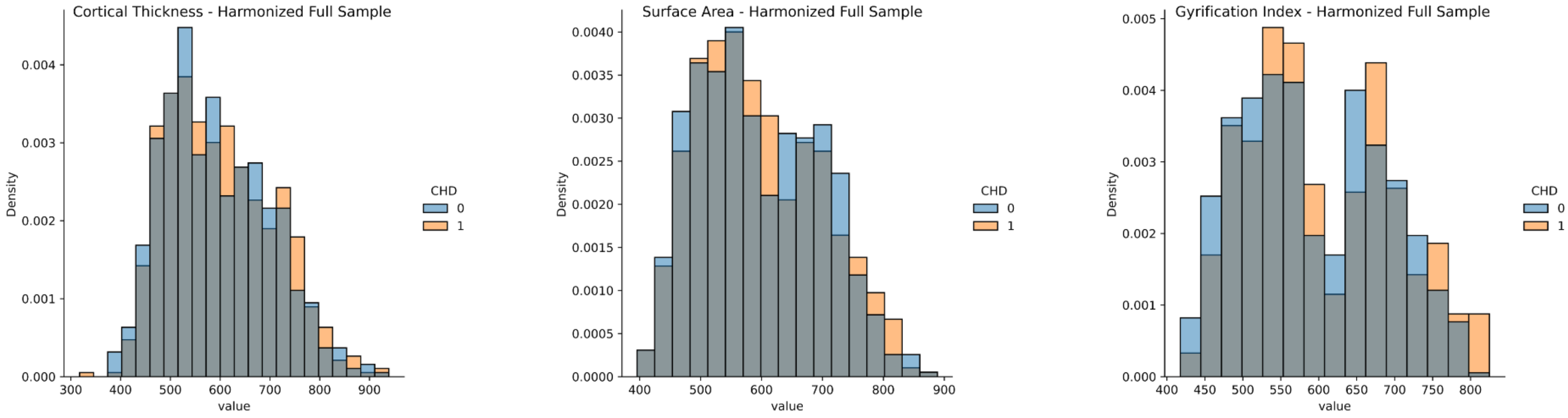

Supplementary Figure 3: Distribution of H-weights per metric.

References

Jubault, T., Gagnon, J.-F., Karama, S., Ptito, A., Lafontaine, A.-L., Evans, A. C., & Monchi, O. (2011). Patterns of cortical thickness and surface area in early Parkinson's disease. *NeuroImage*, *55*(2), 462–467. https://doi.org/10.1016/j.neuroimage.2010.12.043

Kim, J. S., Singh, V., Lee, J. K., Lerch, J., Ad-Dab'bagh, Y., MacDonald, D., Lee, J. M., Kim, S. I., & Evans, A. C. (2005). Automated 3-D extraction and evaluation of the inner and outer cortical surfaces using a Laplacian map and partial volume effect classification. *NeuroImage*, *27*(1), 210–221. https://doi.org/10.1016/j.neuroimage.2005.03.036

Kovacevic, N., Abdi, H., Beaton, D., & McIntosh, A. (2013). Revisiting PLS Resampling: Comparing Significance Versus Reliability Across Range of Simulations. *Springer Proceedings in Mathematics and Statistics*, *56*, 159–170. https://doi.org/10.1007/978-1-4614-8283-3_10

Krishnan, A., Williams, L. J., McIntosh, A. R., & Abdi, H. (2011). Partial Least Squares (PLS) methods for neuroimaging: A tutorial and review. *NeuroImage*, *56*(2), 455–475. https://doi.org/10.1016/j.neuroimage.2010.07.034

Lerch, J. P., & Evans, A. C. (2005). Cortical thickness analysis examined through power analysis and a population simulation. *NeuroImage*, *24*(1), 163–173. https://doi.org/10.1016/j.neuroimage.2004.07.045

MacDonald, D., Kabani, N., Avis, D., & Evans, A. C. (2000). Automated 3-D extraction of inner and outer surfaces of cerebral cortex from MRI. *NeuroImage*, *12*(3), 340–356. https://doi.org/10.1006/nimg.1999.0534

Pedregosa, F., Varoquaux, G., Gramfort, A., Michel, V., Thirion, B., Grisel, O., Blondel, M., Prettenhofer, P., Weiss, R., Dubourg, V., Vanderplas, J., Passos, A., Cournapeau, D.,

Brucher, M., Perrot, M., & Duchesnay, É. (2011). Scikit-learn: Machine Learning in Python. *Journal of Machine Learning Research*, *12*(85), 2825–2830.

Seabold, S., & Perktold, J. (2010). *Statsmodels: Econometric and statistical modeling with python*. *57*(61), 10–25080.

Tohka, J., Zijdenbos, A., & Evans, A. (2004). Fast and robust parameter estimation for statistical partial volume models in brain MRI. *NeuroImage*, *23*(1), 84–97. https://doi.org/10.1016/j.neuroimage.2004.05.007

Toro, R., Perron, M., Pike, B., Richer, L., Veillette, S., Pausova, Z., & Paus, T. (2008). Brain Size and Folding of the Human Cerebral Cortex. *Cerebral Cortex*, *18*(10), 2352–2357. https://doi.org/10.1093/cercor/bhm261

Virtanen, P., Gommers, R., Oliphant, T. E., Haberland, M., Reddy, T., Cournapeau, D., Burovski, E., Peterson, P., Weckesser, W., Bright, J., van der Walt, S. J., Brett, M., Wilson, J., Millman, K. J., Mayorov, N., Nelson, A. R. J., Jones, E., Kern, R., Larson, E., … van Mulbregt, P. (2020). SciPy 1.0: Fundamental algorithms for scientific computing in Python. *Nature Methods*, *17*(3), Article 3. https://doi.org/10.1038/s41592-019-0686-2

Zijdenbos, A., Forghani, R., & Evans, A. (1998). Automatic quantification of MS lesions in 3D MRI brain data sets: Validation of INSECT. In W. M. Wells, A. Colchester, & S. Delp (Eds.), *Medical Image Computing and Computer-Assisted Intervention—MICCAI'98* (pp. 439–448). Springer. https://doi.org/10.1007/BFb0056229

Ziolkowski, J. (2022). Investigating Individual Variability of Morphometric Covariance Patterns in Autism Spectrum Disorder [M.Sc., McGill University (Canada)]. In *ProQuest Dissertations and Theses* (2838440672). Dissertations & Theses @ McGill University; ProQuest Dissertations & Theses Global; ProQuest One Academic.
